# SMA CARNI-VAL TRIAL PART II: A Prospective, Single-Armed Trial of L-Carnitine and Valproic Acid in Ambulatory Children with Spinal Muscular Atrophy

**DOI:** 10.1371/journal.pone.0021296

**Published:** 2011-07-06

**Authors:** John T. Kissel, Charles B. Scott, Sandra P. Reyna, Thomas O. Crawford, Louise R. Simard, Kristin J. Krosschell, Gyula Acsadi, Bakri Elsheik, Mary K. Schroth, Guy D'Anjou, Bernard LaSalle, Thomas W. Prior, Susan Sorenson, Jo Anne Maczulski, Mark B. Bromberg, Gary M. Chan, Kathryn J. Swoboda, for the Project Cure Spinal Muscular Atrophy Investigators' Network

**Affiliations:** 1 Departments of Neurology and Pediatrics, The Ohio State University, Columbus, Ohio, United States of America; 2 CBS Squared, Inc, Fort Washington, Pennsylvania, United States of America; 3 Department of Neurology, University of Utah School of Medicine, Salt Lake City, Utah, United States of America; 4 Department of Pediatrics, University of Utah School of Medicine, Salt Lake City, Utah, United States of America; 5 Departments of Neurology and Pediatrics, Johns Hopkins University School of Medicine, Baltimore, Maryland, United States of America; 6 Department of Biochemistry and Medical Genetics, University of Manitoba, Winnipeg, Manitoba, Canada; 7 Department of Physical Therapy and Human Movement Sciences, Feinberg School of Medicine, Northwestern University, Chicago, Illinois, United States of America; 8 Departments of Neurology and Pediatrics, Wayne State University School of Medicine, Detroit, Michigan, United States of America; 9 Department of Pediatrics, University of Wisconsin School of Medicine, Madison, Wisconsin, United States of America; 10 Division of Pediatric Neurology, Hôpital Sainte-Justine Montréal, Montréal, Québec, Canada; 11 Department of Biomedical Informatics, University of Utah School of Medicine, Salt Lake City, Utah, United States of America; 12 Department of Molecular Pathology, Ohio State University, Columbus, Ohio, United States of America; 13 Primary Children's Medical Center, Salt Lake City, Utah, United States of America; 14 Pediatric Occupational Therapy Services, Chicago, Illinois, United States of America; 15 Department of Pediatrics, University of Utah, Salt Lake City, Utah, United States of America; Brigham and Women's Hospital, Harvard Medical School, United States of America

## Abstract

**Background:**

Multiple lines of evidence have suggested that valproic acid (VPA) might benefit patients with spinal muscular atrophy (SMA). The SMA CARNIVAL TRIAL was a two part prospective trial to evaluate oral VPA and l-carnitine in SMA children. Part 1 targeted non-ambulatory children ages 2–8 in a 12 month cross over design. We report here Part 2, a twelve month prospective, open-label trial of VPA and L-carnitine in ambulatory SMA children.

**Methods:**

This study involved 33 genetically proven type 3 SMA subjects ages 3–17 years. Subjects underwent two baseline assessments over 4–6 weeks and then were placed on VPA and L-carnitine for 12 months. Assessments were performed at baseline, 3, 6 and 12 months. Primary outcomes included safety, adverse events and the change at 6 and 12 months in motor function assessed using the Modified Hammersmith Functional Motor Scale Extend (MHFMS-Extend), timed motor tests and fine motor modules. Secondary outcomes included changes in ulnar compound muscle action potential amplitudes (CMAP), handheld dynamometry, pulmonary function, and Pediatric Quality of Life Inventory scores.

**Results:**

Twenty-eight subjects completed the study. VPA and carnitine were generally well tolerated. Although adverse events occurred in 85% of subjects, they were usually mild and transient. Weight gain of 20% above body weight occurred in 17% of subjects. There was no significant change in any primary outcome at six or 12 months. Some pulmonary function measures showed improvement at one year as expected with normal growth. CMAP significantly improved suggesting a modest biologic effect not clinically meaningful.

**Conclusions:**

This study, coupled with the CARNIVAL Part 1 study, indicate that VPA is not effective in improving strength or function in SMA children. The outcomes used in this study are feasible and reliable, and can be employed in future trials in SMA.

**Trial Regsitration:**

Clinicaltrials.gov NCT00227266

## Introduction

Spinal muscular atrophy (SMA) is an autosomal recessive motor neuron disease that affects approximately 1 in 8,000 newborns. It is a leading cause of infant and childhood morbidity [1]–[6]. The genetics of SMA are complex, but all patients have homozygous mutations in exon 7 of the survival of motor neuron (*SMN1*) gene on chromosome 5q13 [7], [8]. These mutations result in decreased expression of SMN protein, which functions chiefly as part of a complex (the SMN complex) that plays a crucial role in eukaryotic mRNA processing [9]–[11]. SMN protein is also transported in the axon, where it appears to play an important role in neuromuscular junction formation and axonal growth. The relative contribution of these functions to the pathogenesis of SMA is still unclear and a matter of some debate [11].

A feature that makes SMA unique among human genetic diseases is that a genomic duplication at the SMN locus has resulted in a nearly identical gene, *SMN2* that lies centromeric to the *SMN1* gene and differs from *SMN1* mainly by a single C to T nucleotide substitution at the splice junction of exon 7. This mutation does not affect the amino acid sequence, but does alter mRNA splicing in favor of transcripts lacking exon 7 [12]–[17]. A small amount of full-length SMN transcript is produced by the *SMN2* gene, however, and *SMN2* copy number is a major determinant of phenotype [11], [18], [19]. Babies with severe SMA have fewer copies of *SMN2* than those with milder forms of the disease and mouse models of SMA recapitulate this protective effect of *SMN2* copy number on phenotypic severity [9]–[18]. These findings suggest the possibility that pharmacologic or genetic strategies to increase production of full-length transcript from *SMN2* might prove to be an effective therapeutic strategy in SMA.

Valproic acid (VPA) increases SMN expression in SMA patient-derived cell lines as well as in SMA patients probably through its action as a histone deacetylase (HDAC) inhibitor [20]–[26]. VPA has also been shown to improve gross motor function and increase survival time in an SMA mouse model [27], [28]. In these studies, VPA treatment also resulted in larger evoked motor potentials on electrophysiologic studies, less degeneration of spinal motor neurons and improved neuromuscular junction innervation [28]. In addition, three open label trials of VPA in humans all suggested a benefit in strength, motor function, or both [29]–[31]. These encouraging results led us to perform a comprehensive clinical trial of VPA in a large cohort of children with SMA. Because VPA can deplete carnitine stores that are already diminished in SMA patients by low muscle mass, we chose a combined regimen of VPA and carnitine for this study [31], [32]. Part 1 of this trial was a double blind, randomized, intention to treat trial of VPA and carnitine in non-ambulatory SMA patients (CARNI-VAL Part 1) that has previously been reported [32]. We report here the results of CARNI-VAL Part 2, an open-label, single arm trial of VPA and carnitine in ambulatory children with SMA.

## Methods

### Trial Design

The SMA CARNI-VAL trial was a multi-center phase 2 trial of VPA and carnitine in patients with spinal muscular atrophy. The primary objective was to assess the safety, tolerability, and efficacy of a combined regimen of oral VPA and carnitine in SMA patients 2–17 years of age. Secondary objectives were to refine electrophysiological and clinical techniques to better follow the course of ambulatory patients with SMA, in which clinical outcome measures have not been extensively validated. The protocol for this trial and supporting CONSORT checklist are available as supporting information; see Checklist S1 and Protocol S1.

The trial consisted of two parallel multi-center studies, targeting different SMA populations (Clinicaltrials.gov ID NCT00227266) [32]. Part 2 described here was a parallel single-armed, open-label trial in SMA type 2 or 3 “standers and walkers” 3–17 years of age. This open-label trial design was chosen as an initial study for two reasons. First, previous positive studies were small and largely anecdotal in nature, and quality natural history outcomes that could be used to establish power in a randomized clinical trial were lacking. Second, given the availability of VPA and previously reported anecdotal “positive” trials, enthusiasm for a placebo-controlled trial in the relatively small ambulatory SMA community was limited, suggesting that recruitment for a controlled study would likely be extremely difficult. Under these circumstances, we felt that an open label study with objective outcome measures and adverse event ascertainment would improve on the information available and could be completed in a reasonable time frame, with the potential for identifying a possible signal which could be valuable in design of future clinical trials.

### Study population

**Figure 1:**
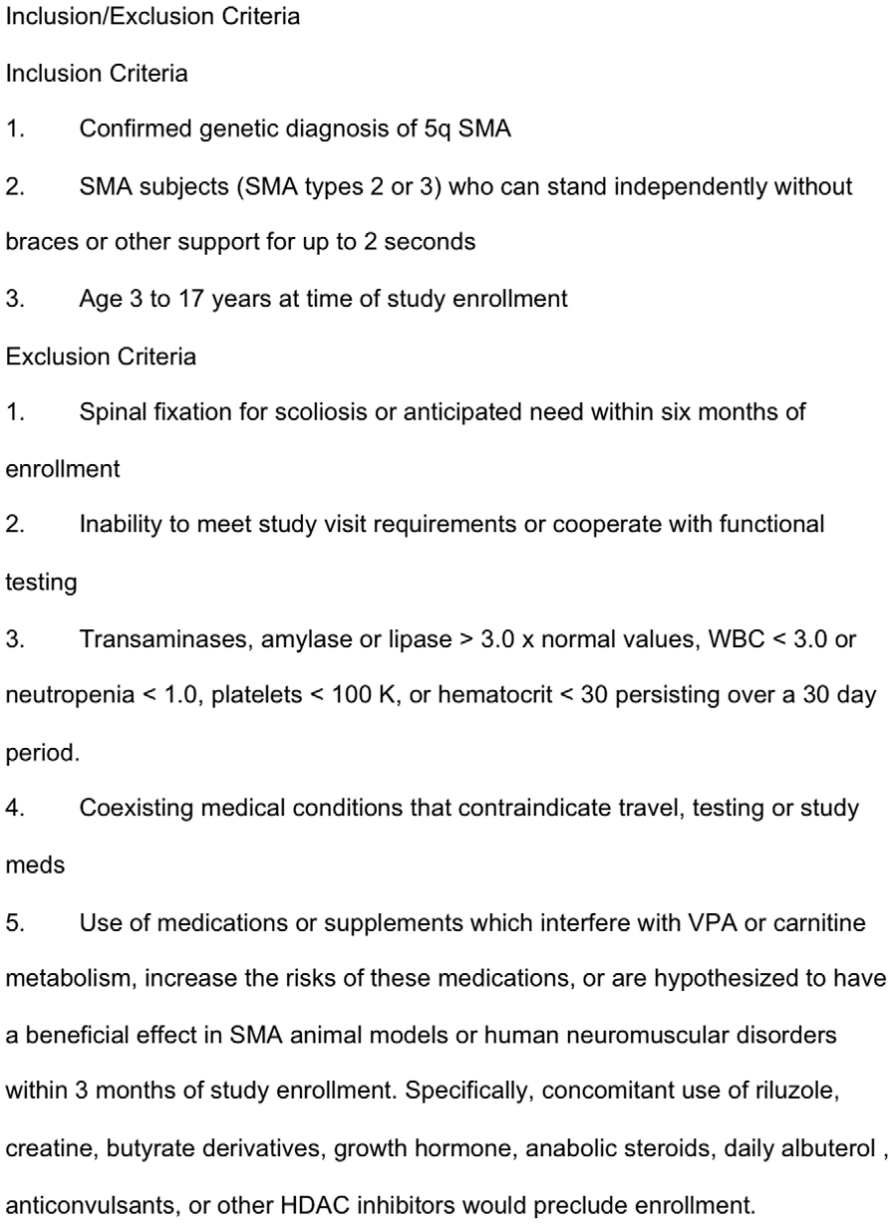
Inclusion/exclusion criteria for study enrollment.

**Figure 2:**
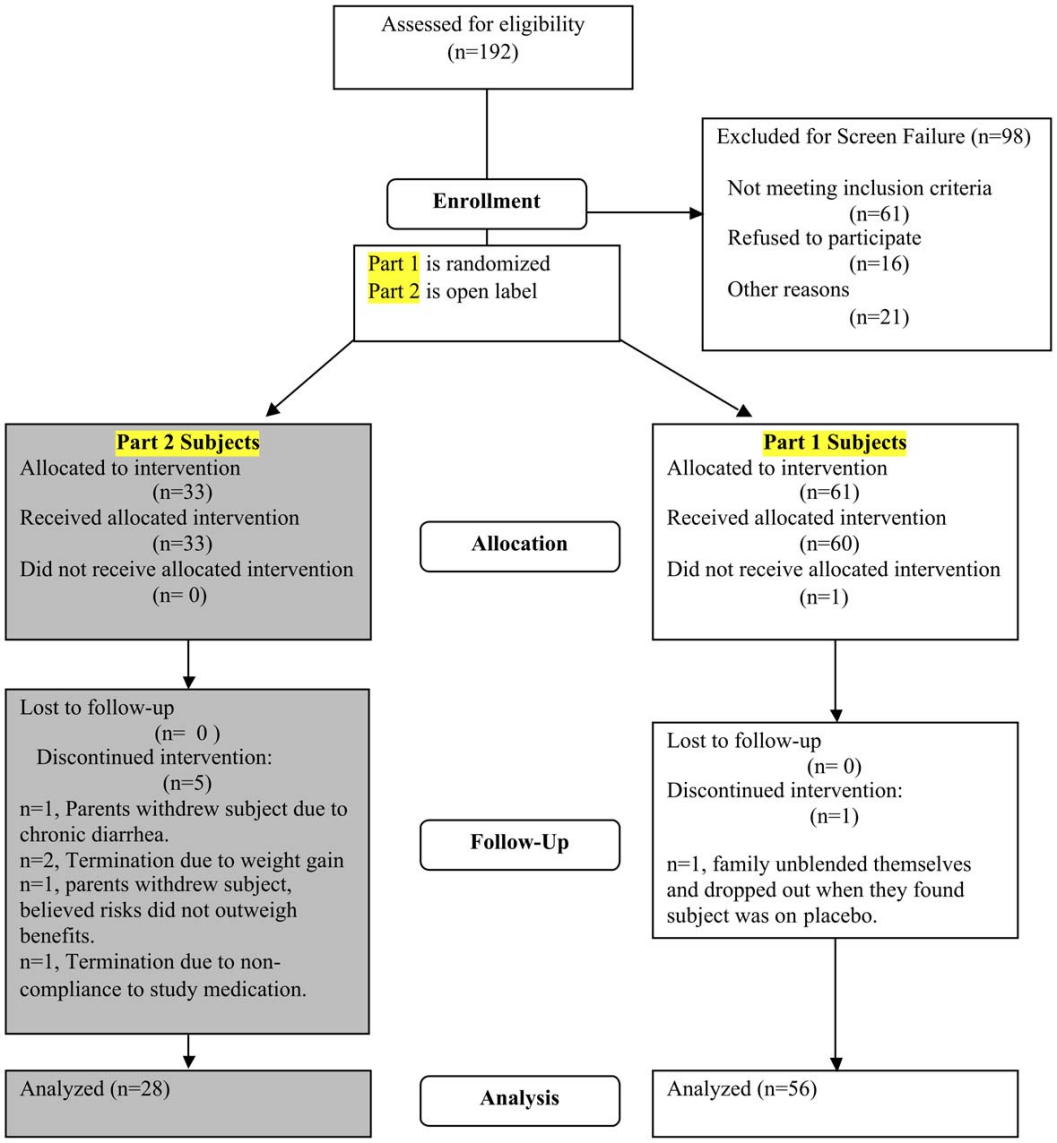
Consort flow sheet.

We prospectively enrolled 33 ambulatory SMA children at six centers in North America following the inclusion and exclusion criteria outlined in Figure 1. This sample size was chosen to accommodate an expected 20% drop out rate and thus provide at least 25 patients completing the study. This sample size provided a 95% confidence interval that would be no wider than plus or minus 40% of the standard deviation around the mean for each functional motor scale measure. The study was approved by the Institutional Review Board (IRB) at each participating clinical trial site (University of Wisconsin-Madison Health Sciences; Wayne State University; Ohio State University; Johns Hopkins University, Centre Hospitalier Universitaire Sainte-Justine, Universite de Montreal, and the University of Utah, the central coordinating site. Written informed parental consent (subjects <18 years) and assent (subjects ≥7 years) were obtained for all subjects. The progress of all participants through the trial for both studies is diagrammed in Figure 2.

### Study procedures

All subjects completed two baseline visits within a six week period to assure that the examiners' methodologies were reliable and that subjects enrolled in the study exhibited test-retest stability prior to the start of the trial. Having two baseline measures also allowed younger subjects to acclimate to testing procedures. Following the second visit, all subjects were placed on VPA and carnitine. All subjects received active treatment for a full 12 months. VPA was provided by Abbott Pharmaceutical as 125 mg divalproex sodium coated particles (Depakote® sprinkle capsules) and L-carnitine was provided by Sigma-Tau Pharmaceutical in a 100 mg/ml liquid (Carnitor®). Divalproex sodium was administered in divided doses two to three times daily to maintain trough levels of 50–100 mg/dL. L-carnitine was dosed at 50 mg/kg/day to a maximum of 1000 mg, divided into two daily doses. Study compliance was assessed through pill counts at each visit with appropriate dosing at least 80% of the time by pill count considered compliance. Compliance was further assessed through trough VPA levels.

Treatment assessments were performed at 3 (V1), 6 (V2) and 12 (V3) months. Safety laboratory studies were performed at baseline, 2–3 weeks following initiation, at each treatment visit and midway between V2 and V3 visits, and included a basic chemistry profile, complete blood count with platelets, transaminases, carnitine profile, amylase, lipase and trough VPA levels. A central medical monitor reviewed all subjects' blood tests and adverse events and performed dosing adjustments or additional testing where necessary. Adverse events were graded using Common Terminology Criteria for Adverse Events v3.0 (CTCAE v3.0). An independent Data and Safety Monitoring Committee provided oversight for the study and performed interim safety data analyses, and had the ability to stop the study if there was a safety concern.

### Outcome measures

Primary outcome measures included laboratory safety and adverse event data, as well as efficacy as measured by change from baseline at 6 and 12 months in the Modified Hammersmith Functional Motor Scale-Extend (MHFMS-Extend), timed tests of function (TTF) and fine motor modules (FMM). The MHFMS-Extend includes the 20 items in the original MHFMS, as well as an additional eight items in a Gross Motor Module (GMM). Details of this testing and the appropriate protocols are available at http://smaoutcomes.org [33]. For statistical purposes, each component of the testing was analyzed individually as discussed below.

Secondary outcome measures, as in the Part 1 study, included maximum ulnar compound muscle action potential (CMAP) amplitude as an estimate of innervation, dual-energy X-ray absorptiometry (DEXA) evaluation of body composition and bone density, quantitative assessment of SMN mRNA; evaluation of quality of life using the Pediatric Quality of Life Inventory (PedsQL™) and for children >5 years, change from baseline measures of pulmonary function and muscle strength via handheld myometry at 6 and 12 months [31], [32]. The protocol for ulnar CMAP amplitude determination has been previously described and is available at http://smaoutcomes.org. Dual-energy X-ray absorptiometry (DEXA) scanning for bone density and body composition was performed at the Columbus, Salt Lake City, and Madison sites using the Norland DEXA XR-36 software version 3.3.1 for small subjects. Relative quantification of full-length (flSMN) and exon 7-lacking (Δ7 SMN) SMN transcripts in whole blood was performed as previously described [32]. Results are reported as relative amounts of flSMN or Δ7 SMN normalized against RPLPO (large ribosomal protein). Quality of life (QOL) was assessed using the 0–100 scale (PedsQL™) [34]. The same parent completed the (PedsQL™) at each visit and children >5 years of age completed the age-appropriate (PedsQL™). A change of 4.4 in the child self-report and 4.5 in the parent-proxy report was considered a meaningful difference in this instrument [34]. Pulmonary function testing (PFT) was also feasible only in children ≥5 years and included forced vital capacity (FVC), forced expiratory volume in 1 second (FEV1) and maximum expiratory and inspiratory pressures (MEP, MIP). Myometry measurements (done only in children ≥5 years of age) were performed three times for right and left elbow flexion, and for right and left knee extension, at each visit using the Lafayette Instrument MMT System Model 01163 myometer [31], [32], [35]–[37], with the recorded value representing the average of the three measures.

### Statistical Analysis

Two baseline visits were performed with the visit closest to the start of treatment used as the baseline evaluation for outcome variables. Shapiro-Wilk test was used to determine if continuous variables were normally distributed. Test-retest correlation used the Spearman's correlation if continuous variables were not normally distributed. Timed tests had a restricted maximum of 3 minutes at which time the test was ended. Since the actual maximum time to complete the task was unknown, we considered these values as censored. Kaplan-Meier estimates were used to compute the summary statistics for timed tests with censored observations. Statistical tests for change from baseline were either the Wilcoxon signed rank test for non-normal data or the paired t-test for normal data. Change from baseline analyses to each time point were selected to identify if a 6 month change occurred, a 12 month change occurred, or there was no change. The objective of this analysis was to evaluate patterns in the data and not provide definitive hypothesis testing; therefore, no multiplicity adjustment was performed.

## Results

Thirty-three subjects were enrolled with demographic characteristics shown in Table 1. The subjects were ages 2.8 to 16.3 with a median age of 6.9. There was an unexpected predominance of males (n = 22). Five patients did not complete the study; two because of weight gain considered excessive by the parents (9% increase above baseline weight in each case), one because of gastrointestinal side effects (chronic diarrhea), one because the parents simply changed their minds about participation, and one because of non-compliance and psychiatric issues that predated participation in the study and were not disclosed to investigators at the time of screening and randomization (Figure 2). This subject subsequently missed several study visits, was found to be non-compliant with study medications, and therefore was withdrawn from the study. A sixth patient missed the V1 visit but all other data points were included in the final analysis. All of the subjects completing the study were judged compliant by both pill counts and by VPA levels.

**Table 1:** Patient Demographics.

Characteristic	N = 33	(%)
Age (years):		
Median	6.9	
Range	2.8–16.3	
Gender:		
Female	11	(33.3)
Male	22	(66.7)
Ethnicity:		
Hispanic	0	(0.0)
Non-Hispanic	30	(90.9)
Unknown	3	(9.1)
Race:		
Asian	1	(3.0)
African American	0	(0.0)
White	29	(87.9)
Unknown	3	(9.1)

### Adverse Events

Adverse events (AEs) occurred in 84.8% of treated subjects during treatment, a figure not significantly different from the side effects encountered in the treatment and placebo group of the Phase 1 study [32]. A detailed alphabetical listing of adverse events with Common Terminology Criteria for Adverse Events v3.0 (CTCAE) grading is presented in Table 2. General systemic disorders (e.g. fatigue, fever, flu-like symptoms, irritability, pain) were the most frequent AE (36.5% of total AEs), followed by various infections (24.2% each). Only 5 AEs (4% of total) were classified as CTCAE Grade 3 (severe); all of these were transient and did not require study withdrawal. Three patients developed adverse events that led to withdrawal from the study; one had chronic diarrhea, and two had weight gain deemed unacceptable by the parents. Weight gain was a notable problem although interestingly most parents and investigators did not consider this an adverse event; 17% of the patients that completed the study gained 20% or more of baseline weight, and one subject's weight increased by 49% in one patient.

**Table 2:** Treatment-related Adverse Events (AE).

CTCAE System Organ Class/Preferred Term (MedDRA)	N (%) Tot. AE	Grade 1	Grade 2	Grade 3
**Blood/Lymphatic System Disorders:**	**1 (0.7)**			
Blood/lymphatic disorder	1			1
**Ear and Labyrinth Disorders:**	**2 (1.5)**			
Hearing impaired	1		1	
Middle ear inflammation	1	1		
**Gastrointestinal Disorders:**	**15 (10.9)**			
Abdominal pain	5	3	1	1
Constipation	1	1		
Diarrhea	1		1	
Vomiting	6	5	1	
Other	2	2		
**General Disorders:**	**50 (36.5)**			
Fatigue	17	14	2	1
Fever	2	1	1	
Flu like symptoms	4	3	1	
Irritability	4	3	1	
Pain	5	2	1	2
Other	18	8	10	
**Immune System Disorders:**	**2 (1.5)**			
Allergic reaction	2	1	1	
**Infections and Infestations:**	**24 (24.2)**			
Eye infection	1		1	
Otitis media	4	2	2	
Rhinitis infective	3	3		
Sinusitis	2		2	
Skin infection	1	1		
Upper respiratory infection	1		1	
Other	12	12		
**Injury, Poisoning and Procedural Complications:**	**5 (3.6)**			
Fall	1		1	
Fracture	4	1	3	
**Investigations:**	**1 (0.7)**			
Neutrophil count decreased	1	1		
**Metabolism and nutrition disorders:**	**1 (0.7)**			
Dehydration	1	1		
**Musculoskeletal and connective tissue disorders:**	**2 (1.5)**			
Pain in extremity	1	1		
Other	1	1		
**Nervous System Disorders:**	**6 (4.4)**			
Headache	1	1		
Lethargy	2	1	1	
Tremor	3		3	
**Psychiatric disorders:**	**4 (2.9)**			
Mania	1		1	
Restlessness	1		1	
Other	2	1		1
**Respiratory, thoracic and mediastinal disorders:**	**19 (13.9)**			
Allergic rhinitis	1	1		
Cough	7	5	2	
Nasal congestion	4	3	1	
Pneumonitis	4	4		
Sore throat	1	1		
Other	2	1	1	
**Skin and subcutaneous tissue disorders:**	**5 (3.6)**			
Rash maculo-papular	2	1	1	
Other	3		3	

Events are listed alphabetically with percentages indicating percent of total AEs. Grading is according to standard Common Terminology Criteria for Adverse Events (CTCAE) grading.

### Reliability of the motor function scores

The average MHFMS-Extend score at baseline was 48.3 (±5.4 S.D.) with a range of 36 to 56 (median 48). The MHFMS-Extend baseline data was skewed left (Shapiro-Wilk p = 0.048). The test-retest reliability of the MHFMS-Extend, assessed for S1-S2, was 0.93 (Table 3).

**Table 3:** Test-retest Reliability of the MHFMS-Extend S1 vs. S2.

	N	Minimum	Median	Maximum
S1 MHFMS-Extend	30	29	48	56
S2 MHFMS-Extend	30	36	48	56
	Spearman's correlation 0.9307			

**Figure 3:**
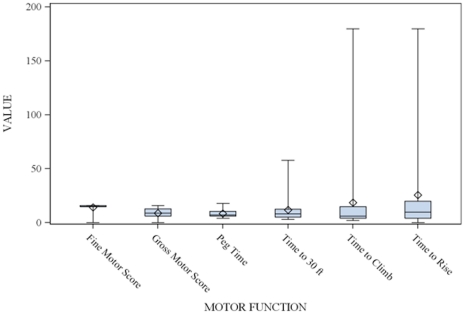
Box Plots of baseline values for Fine Motor Module, Gross Motor Module, and Timed Tests of Function. The Values on the ordinate refer to seconds for the timed tests, and absolute scores for the motor modules. Δ indicates mean; – indicates median.

The baseline scores for the TTF, GMM component of the MHFMS-Extend, and FMM are presented in Figure 3; all of the measures were skewed and not normally distributed. Table 4 presents the Spearman correlation for each TTF, GMM, and FMM scores to assess test-retest reliability. The data indicate excellent reliability for all measures of motor function except time to rise.

**Table 4:** Reliability (Test-Related) of Timed Tests and Tests of Motor Function.

Item Score	Median (1^st^ Baseline)	Median (2^nd^ Baseline)	Spearman Correlation	p-Value
Time to 30 ft	8.5	8.0	0.9734	<0.0001
N = 28				
Time to Climb	5.5	6.0	0.9190	<0.0001
N = 24				
Time to Rise	9.5	8.5	0.7240	<0.0001
N = 24				
Peg Time	9.0	7.0	0.8600	<0.0001
N = 29				
Fine Motor	15.5	15.0	0.8272	<0.0001
N = 30				
Gross Motor	8.0	9.5	0.9267	<0.0001
N = 30				

### Impact of treatment on timed tests and gross motor function

Tables 5 and 6 illustrate the absolute scores at each time point and the change from baseline at 6 months and 12 months for TTF, FMM, GMM and total MHFMS-Extend. A change from baseline score less than zero for the timed scores (i.e. a negative number) reflects reduced time to perform the function and thus improvement. Motor scores that have a positive change from baseline (i.e. positive numbers) indicate improved function. There was no significant change in function as assessed by timed test and motor function at either 6 or 12 months.

**Table 5:** Timed Tests and Motor Function; 6 Month Observed Values and Change from Baseline.

Item	6 Month Value	Change from Baseline
Time to 30 ft		
N	26	26
Median	7.5	0
Range	3–39	−5–17
Time to Climb		
N	25	23
Median	7	−1
Range	2–47	−26–2
Time to Rise		
N	25	25
Median	9	0
Range	2–55	−146–29
Peg Time		
N	27	26
Median	8	0
Range	4–17	−4–4
Fine Motor Score		
N	28	28
Median	15.5	0
Range	0–16	−10–15
Gross Motor Score		
N	28	28
Median	8	1
Range	0–16	−5–3
MHFMS-Extend		
N	28	28
Median	48	1
Range	32–56	−11–7

**Table 6:** Timed Tests and Motor Function 12 Month; Observed Value and Change from Baseline.

Item	12 Month Value	Change from Baseline
Time to 30 ft:		
N	25	24
Median	7	0
Range	3–180	−3–49
Time to Climb:		
N	22	21
Median	6.5	0
Range	2–180	−51–7
Time to Rise:		
N	22	22
Median	8	0
Range	2–180	−12–45
Peg Time:		
N	26	25
Median	8.5	0
Range	4–18	−9–3
Fine Motor Score:		
N	27	27
Median	15	0
Range	0–16	−16–15
Gross Motor Score:		
N	27	27
Median	8	0
Range	0–16	−5–3
MHFMS-Extend:		
N	27	27
Median	48	0
Range	30–56	−13–4

### Myometry

**Figure 4:**
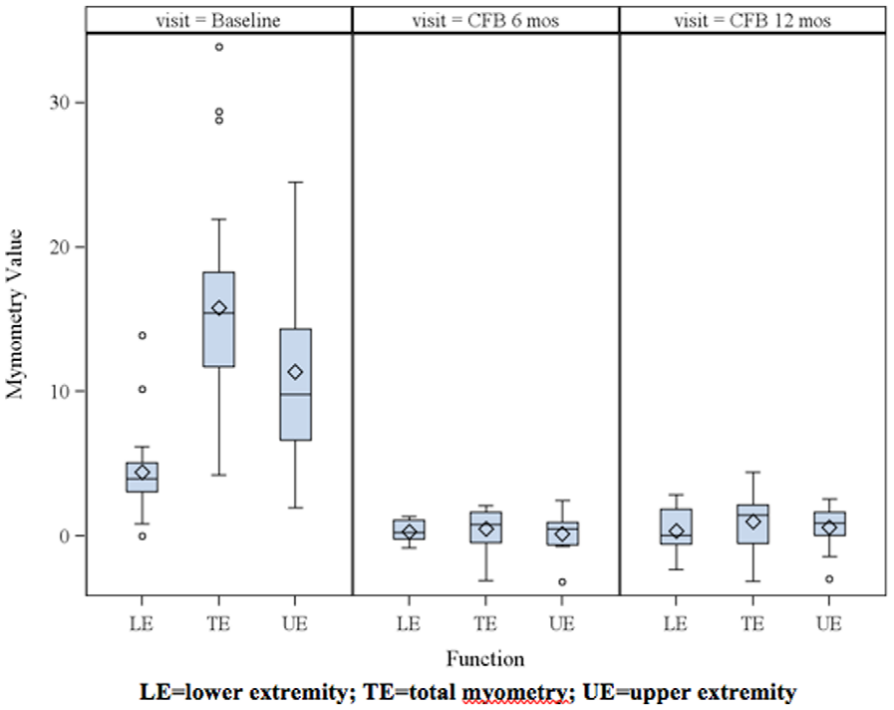
Box plot of baseline myometry data (column 1) and change from baseline (CFB) at 6 months (column 2) and 12 months (column 3). LE = lower extremity; TE = total myometry; UE = upper extremity. Δ indicates mean; – indicates median.

Because of age restrictions (≥5 years) and limitations from contractures, only 20 subjects had baseline evaluations and only 11 had a subsequent myometry assessment at 6 months. The upper extremity and total myometry scores were normally distributed, but the lower extremity was skewed. The baseline myometry data and change at 6 and 12 months are shown in Figure 4. There were no statistical differences in myometry scores over the course of the study.

### Treatment effects on electrophysiologic measures of innervation

The results for CMAP testing at baseline and 6 and 12 months, as well as the change from baseline to these time points, are shown in Table 7. There was a statistically significant improvement in CMAP negative peak amplitude at six and 12 months.

**Table 7:** Change from Baseline (CFB) at 6 and 12 Months in Compound Motor Action Potential (CMAP).

	Baseline	CFB 6 Months	CFB 12 Months
Characteristic	N = 24	N = 20	N = 13
CMAP Amplitude:			
Median	5.30	0.70	1.66
Range	1.20–10.42	−0.80–3.00	−1.40–4.55
Shapiro-Wilk	p = 0.2928		
Paired t-test		p = 0.0022	p = 0.0012

### Impact of treatment on quality of life outcome assessments

Quality of life was measured using the PedsQL™, both as reported by parent-proxy and, where appropriate, by the subjects themselves. The baseline self-reported PedsQL™ data and change at six and 12 months are shown in Table 8. According to the child's own assessment, there was a deterioration of physical functioning at 12 months (p = 0.008). There was no associated change in any domain of quality of life by parent assessment (data not shown).

**Table 8:** Child Self-assessed PedsQL; Change from Baseline (CFB) at 6 and 12 Month.

	Baseline	CFB 6 Months	CFB 12 Months
Characteristic	N = 22	N = 17	N = 16
Physical Function:			
Median	60.9	−6.2	−3.1
Range	31.2–93.7	−31.2–18.7	−31.3–6.3
Shapiro-Wilk	0.1862		
Paired t-test		0.0974	0.0080
Emotional Function:			
Median	70	0	10
Range	45–100	−30–30	−30–30
Shapiro-Wilk	0.0118		
Social Function:			
Median	70	0	2.5
Range	25–85	−20–40	−30–30
Shapiro-Wilk	0.0128		
School Function:			
Median	80	−5	−2.5
Range	40–95	−15–10	−35–15
Shapiro-Wilk	0.0003		
Psychosocial:			
Median	71.7	0	3.3
Range	46.7–93.3	−16.7–11.7	−20–20
Shapiro-Wilk	0.0556		
Total QOL:			
Median	67.4	0	0
Range	48.9–87	−17.4–7.6	−19.6–8.7
Shapiro-Wilk	0.2733		

### Pulmonary Function Testing

The PFT did not depart from normality at baseline. None of the PFT parameters changed significantly from baseline at six months (data not shown). At one year, only FVC (p = 0.04) and FEV1 values (p = 0.009) had significantly improved, a change that must be interpreted with caution, since FVC normally increases normally with age [38].

### VPA Trough Levels

Table 9 presents VPA trough levels. On average, subjects achieved the desired trough level of 50, providing strong evidence of compliance with the regimen. There were no associations with VPA levels and timed tests or MHFMS-Extend.

**Table 9:** Valproic Acid Trough Levels by Visit.

Visit	Number	Mean	Std Dev	Minimum	Median	Maximum
V2	25	59.9	22.4	18.3	60.0	120.1
V3	23	66.1	23.4	31.9	60.3	121.9

### mRNA Assay

The baseline and change from baseline at 6 and 12 months for flSMN and Δ7 SMN transcripts are shown in Table 10. There was no significant change in SMN transcript level from baseline at either time point.

**Table 10:** Mean normalized relative amount of SMN mRNA at 6 and 12 Months Actual Values and Change from Baseline (CFB) in full length (fl) and Δ7SMN.

		6 Months	12 Months
flSMN	N	25	20
	Mean	2.663	2.777
	Std Dev	0.804	0.852
	Min	0.760	0.734
	Max	3.842	4.362
Δ7 SMN	N	25	20
	Mean	6.267	6.266
	Std Dev	1.511	1.334
	Min	1.455	4.121
	Max	8.154	8.866
CFB flSMN	N	24	19
	Mean	−0.101	0.073
	Std Dev	0.363	0.169
	Min	−1.688	−0.281
	Max	0.199	0.545
CFB Δ7 SMN	N	24	19
	Mean	−0.173	0.095
	Std Dev	1.019	0.722
	Min	−2.680	−1.169
	Max	1.275	1.487

## Discussion

The combination of VPA and L-carnitine did not lead to improvement in the primary outcome variables under the conditions defined by this protocol in an ambulatory population of children with SMA. The primary outcome measure of the MHFMS-Extend proved reliable and practical, and experience with many of the secondary outcome variables employed in this study will prove useful in the design of future clinical trials in this group. These results in this open-label trial further establish the reliability of these measures for clinical trials. The combination of VPA and L-carnitine proved safe at the doses used. Among the secondary outcomes, myometry measured strength and quality of life also failed to show any improvement. Although some measures of pulmonary function (FVC and FEV1) did show significant improvement at one year, these changes are within the range expected with normal growth in children with SMA, for which natural history data are limited; these findings are therefore difficult to interpret especially given the other negative results. CMAP amplitude did improve at six and 12 months, but without a corresponding increase in function, the significance of this finding is difficult to interpret. However, it is possible that a modest biologic effect on sprouting was negated by weight gain, voiding any ultimate impact on motor functions. The lack of a significant change in relative SMN transcript levels after 6 and 12 months is consistent with the absence of a VPA effect on clinical measures. A more effective treatment is needed before we can adequately assess the value of quantifying SMN transcripts in whole blood as a potential surrogate marker of drug response.

We did not observe any clinical or laboratory evidence of serious hematologic or hepatic toxicity in this study, but four patients dropped out due to medication side effects. Excessive weight gain was a common adverse event that was almost certainly compounded by treatment with VPA. However, children and young adults with ambulatory SMA who are not on VPA are also prone to excessive weight gain with age, and this needs to be carefully considered in the design of future clinical trials, since this clearly plays a role in functional decline with age in some ambulatory children. DEXA scanning revealed that the associated weight gain was due largely to an increase in total body fat mass in the absence of an increase in lean mass (data not shown), an obvious concern in a population already predisposed to higher fat mass indices or frank obesity [38], [39]. Although considerable in some patients in this study, however, the weight gain did not appear to have deleterious effects on functioning in this population in the context of this study over the time period examined.

Both this and the companion study on non-ambulatory children ages 2 to 8 failed to demonstrate improvement in the primary outcome variable [31]. The choices of inclusion criteria, dose, duration, and outcome variables were necessarily based on our best hypotheses given the lack of previous studies with VPA in children with SMA, and limited experience in rigorous clinical studies in this population. However, the experience gained in this trial will be invaluable to the design of future trials. Whether a treatment effect of VPA exists in SMA under specific circumstances, and can be demonstrated in trials in other targeted groups remains possible and hints of this are suggested by subgroup analysis of the non-ambulatory children. At least in this trial, however, any modest biologic effects were likely negated by weight gain in terms of any ultimate effect on motor function. Studies on the efficacy of VPA in ambulatory adults and a preliminary study of VPA safety in severely affected infants are ongoing. These studies will provide additional valuable information to guide us regarding the most appropriate clinical trial designs and choice of primary outcome measures as more potent therapies become available.

## Supporting Information

Checklist S1 (DOC)

Protocol S1 (DOC)
